# Clinical course of patients with severe COVID-19 pneumonia treated with remdesivir: A real-life study

**DOI:** 10.1371/journal.pone.0267283

**Published:** 2022-04-28

**Authors:** Diana Tejada, Regina Juanbeltz, María Rivero, Ramón San Miguel, Ferrán Capdevila, Juan José Beloqui, Maite Sarobe

**Affiliations:** 1 Department of Hospital Pharmacy, Hospital Universitario de Navarra, Pamplona, Navarra, Spain; 2 Navarre Institute for Health Research (IdiSNA), Pamplona, Navarra, Spain; 3 CIBER Epidemiología y Salud Pública (CIBERESP), Pamplona, Navarra, Spain; 4 Infectious Diseases Unit, Complejo Hospitalario de Navarra, Pamplona, Navarra, Spain; 5 Centro de Investigación Médica Navarrabiomed, Pamplona, Navarra, Spain; Bari University Aldo Moro, ITALY

## Abstract

**Background:**

There is currently much uncertainty regarding the most optimal treatment for COVID-19. This study analyze the change in the clinical condition of patients hospitalized for severe COVID-19 pneumonia and treated with remdesivir in a real-life setting, based on the WHO Ordinal Scale. Clinical complications, treatment safety, and impact of other associated drugs were also analyzed.

**Methods:**

We conducted an observational, retrospective study including patients treated with remdesivir. The need for admission to the ICU, the length of ICU and hospital stay, and the need for ventilatory support were analyzed. The laboratory parameters, drugs administered concomitantly, and difference in the length of hospital stay according to the concomitant treatment received were also evaluated. A univariate and multivariate Cox regression analysis was performed to analyze associated factors.

**Results:**

A total of 92 patients were included. The mean length of hospital stay was 15 days, and 90% of the patients had been discharged from the hospital 28 days after starting treatment with remdesivir. The likelihood of hospital discharge among patients not presenting with hypertension as a comorbidity was significantly higher than that of those with this condition (HR = 3.19, P = 0.008). Nineteen patients had to be admitted to the ICU (mean of 18 days). Approximately 11% required invasive mechanical ventilation (mean of 22 days). Almost 37% of the patients received high-flow oxygen therapy and 14% non-invasive mechanical ventilation. Four deaths were recorded within the first week. Main adverse events were increases in transaminase and creatinine levels. Nosocomial infections were more frequent when remdesivir was combined with immunosuppressive drugs.

**Conclusions:**

Patients with severe COVID-19 pneumonia and treated with remdesivir require relatively prolonged hospital stays, many with a need for ventilatory support and, in a considerable proportion of cases, admission to the ICU. However, the observed survival rate is high, and the drug is well tolerated.

## Introduction

The disease caused by the severe acute respiratory syndrome coronavirus 2 (SARS-CoV-2), also known as the 2019 coronavirus disease (COVID-19), was first detected in Wuhan, China, in December 2019 and declared a pandemic on 11 March 2020 by the World Health Organization (WHO).

Its clinical manifestations are unclear and highly variable, with most people developing a mild to moderate disease (80%). However, 15% of the population may progress to a severe disease and 5% could develop complications such as respiratory failure, acute respiratory distress syndrome (ARDS), thromboembolism, septic shock, or multiorgan failure [1]. Validation of markers of disease severity is crucial to identify COVID-19 patients who would benefit from a close monitoring and intensive care.

Remdesivir (RDV) is an adenosine analog prodrug that inhibits viral ribonucleic acid polymerase and has demonstrated antiviral activity both *in vitro*, against a large number of RNA viruses, including SARS-CoV-2 [2], and *in vivo*, in animal models of SARS-CoV-2 infection [3, 4].

It is the first antiviral agent authorized in the European Union for the treatment of COVID-19, with its conditional marketing authorization having been granted in July 2020.

Given the high demand and limited initial availability of the drug, in September 2020, the Spanish Agency of Medicines and Medical Devices (AEMPS, *Agencia Española de Medicamentos y Productos Sanitarios*) defined a pharmaco-clinical protocol for the use of RDV in Spain, in which it authorized its administration to adults and adolescents ≥12 years, weighing ≥40 kg, with severe pneumonia requiring low-flow oxygen therapy (LFO), a maximum time since symptom onset of seven days, and at least two of the following criteria: respiratory rate (RR) ≥24 bpm, oxygen saturation (O_2_Sat) <94%, or a ratio of arterial partial pressure of oxygen to fraction of inspired oxygen (PaO_2_/FiO_2_) <300 mmHg. The most critical patients requiring high-flow oxygen therapy (HFO), non-invasive mechanical ventilation (NIV), invasive mechanical ventilation (IMV), or extracorporeal membrane oxygenation (ECMO) were specifically excluded from the pharmaco-clinical protocol. Patients with alanine aminotransferase (ALT) or aspartate aminotransferase (AST) levels ≥5 times the upper limit of normality (ULN), severe renal failure (defined by a glomerular filtration rate [GFR] <30 ml/min), undergoing hemodialysis or peritoneal dialysis, pregnant women, and those with signs of multiorgan failure were also excluded from the national protocol [5]. The AEMPS recommended a five-day treatment course based on the results of a study revealing no benefit associated with prolonged treatment [6].

COVID-19 has triggered a devastating global health crisis, causing enormous loss of lives. Since the beginning of the pandemic, multiple clinical trials of drugs have been conducted worldwide. However, there is still much uncertainty regarding the most optimal treatment for COVID-19.

In vitro activity of RDV must be extrapolated to clinical practice, confirming its benefit in patients. The results of clinical trials performed to date with RDV are unclear and inconclusive [6–9] andhere is also still little real-life evidence on the effectiveness and safety of treatment with this antiviral agent. Our real-life study was carried out in a Spanish tertiary hospital during the pandemic period, from 3 July 2020 to 31 October 2020. The primary objective of the study was to analyze the change in the clinical condition of patients hospitalized for severe COVID-19 pneumonia and treated with RDV in a real-life setting, based on the modified six-point WHO Ordinal Scale for Clinical Improvement (1 = not hospitalized; 6 = death) [10]. Its secondary objectives were to describe the clinical complications, treatment safety, and impact of other associated drugs, such as biologics and corticosteroids.

## Materials and methods

### Design and study population

An observational, retrospective study was conducted in a regional reference hospital in northern Spain (Hospital Complex of Navarra).

The study considered all patients 12 years of age or older, hospitalized with a severe COVID-19 pneumonia [5], and who had received at least one dose of RDV for the treatment of the disease according to the center’s treatment protocol.

All patients had a SARS-CoV-2 infection confirmed by reverse-transcription polymerase chain reaction (RT-PCR) or a positive nasopharyngeal swab antigen test. Only pregnant women and children aged <12 years were excluded from the study, as these patients were granted access to the drug through a specific compassionate use program.The inclusion period was four months. It elapsed between the moment the conditional marketing authorization for RDV was granted by the European Medicines Agency (EMA), on 3 July 2020, and 31 October 2020.

### Assessments

Prior to starting this study, a revision of the published evidence of COVID-19 treatment with RDV was made. Data available since the start of the pandemic (February 2020) to November 2020 was searched in PubMed database, using the terms: (remdesivir [Title/Abstract]) AND (COVID-19 [Title/Abstract]) OR ((SARS-CoV-2 [Title/Abstract]) OR (severe acute respiratory syndrome coronavirus 2 [Title/Abstract])). To complete the evidence, data related to RDV efficacy and security was also requested from the pharmaceutical company. Studies related to drug mechanism were excluded. Both clinical trials and real-life studies published in any language were searched, focusing on efficacy and security outcomes. Studies regarding the initial access for RDV through the compassionate use program were also considered, as those were the few real-life studies available at the beginning of the pandemic.

Demographic data (age and sex) and clinical variables were obtained from the patients’ computerized medical records (CMRs): comorbidities, signs and symptoms on admission and at the beginning of treatment with RDV, laboratory parameters (on days 0, 3, 5, 14, and 28 after the beginning of treatment with RDV), and concomitant drugs used in the context of the therapeutic strategy for the treatment of COVID-19 were analyzed.

Infectious complications were considered as any in-hospital positive culture requiring medical treatment. All adverse events were classified according to the Common Terminology Criteria for Adverse Events (CTCAE), version 5.0, of the National Cancer Institute (NCI) [11].

Data relating to the need for admission to the intensive care unit (ICU), the length of ICU and hospital stay, and the need for ventilatory support (LFO, HFO, NIV, IMV/ECMO) were also extracted from the patients’ CMRs and analyzed.

### Outcomes

The primary endpoint was the change in the clinical condition of the hospitalized patients 7 and 28 days after starting treatment with RDV, based on the validated modified six-point WHO Ordinal Scale for Clinical Improvement, where 1 = not hospitalized, 2 = hospitalization without supplemental oxygen therapy, 3 = LFO, 4 = HFO/NIV, 5 = ECMO or IMV, and 6 = death [10].

The secondary endpoints were mortality, adverse events associated with RDV and clinical complications (measures reported by clinicians in the patients’ CMRs). All-cause mortality and the causes of death were analyzed during a 42-day follow-up period.

### Statistical analysis

The mean and standard deviation (SD) or the median and interquartile range (IQR) were used in the descriptive analysis of continuous variables depending on whether or not the variables followed a normal distribution, respectively. Categorical variables were expressed as frequencies and percentages.

Differences between patient groups were analyzed using Student’s t-test for continuous variables with a normal distribution and the Wilcoxon test for continuous variables that did not follow such distribution. In the case of categorical variables, the comparison was carried out using the Chi-square or Fisher’s exact test.

Due to the retrospective and observational design of the study, patients often received multiple drugs for the treatment of COVID-19 according to clinical practice. In order to compare whether there were differences in the clinical evolution depending on the different treatments received, a stratified analysis was made by subgroups: RDV ± biological treatment (tocilizumab and/or anakinra) ± corticosteroid boluses, and RDV in monotherapy or associated with low-dose corticosteroid therapy.

A Kaplan-Meier analysis was performed using a log-rank test to determine whether there were differences in the length of hospital stay depending on the treatment administered concomitantly with RDV. In addition, a univariate and multivariate Cox regression analysis was also performed to analyze associated factors. The proportional hazard assumption was checked graphically with the plot of the Schoenfeld residuals and complemented with the assessment of the residuals autocorrelation.

A p-value <0.05 was considered statistically significant.

The statistical analysis was carried out with the IBM SSPS statistical package (version 25).

### Ethical aspects

The study was authorized by the hospital’s Ethics Committee (code PI2020/145) and carried out in accordance with the ethical principles set forth in the Declaration of Helsinki and the Good Clinical Practice guidelines.

Furthermore, the study was approved by the administrative department of both the hospital and the Regional Health Service (Navarra Health Service).

By means of Resolution number 1387/2017 dated on the 8th of November 2020, the manager of the Navarra Health Service establishes an extraordinary procedure in order to authorize the access of clinical data for any public interest research studies when a written informed consent is not feasible. According to this Resolution, the Ethics Committee waived the need for consent from both patients and parents or guardians of the minors candidate to this study.

## Results

### Patients’ demographic and clinical characteristics

Ninety-two patients who had received at least one dose of RDV throughout the study period were included in the study. The demographic and clinical characteristics of these patients are summarized in Table 1. The mean age of the patients was 58 years (SD = 14.3), with 48 (52.2%) being male and 44 (47.8%) female. Half of the studied cohort had between one and two comorbidities, with obesity (35.9%), hypertension (29.3%), and diabetes (14.1%) being the most prevalent.

**Table 1:** Patients’ demographic and clinical characteristics.

	Total	Group 1^a^	Group 2^b^	P value
	n = 92	n = 73	n = 19	
**Age, mean (SD)**	58.3 (14.3)	58.6 (14.3)	56.9 (14.5)	0.637
**Sex, n (%)**				0.964
**Male**	48 (52.2)	38 (52.1)	10 (52.6)	
**Female**	44 (47.8)	35 (47.9)	9 (47.4)	
**Country of birth, n (%)**				0.387
**Spain**	50 (54.3)	38 (52.1)	12 (63.2)	
**Other**	42 (45.7)	35 (47.9)	7 (36.8)	
**No. of comorbidities, n (%)**				**0.017**
**0**	34 (37.0)	22 (30.1)	12 (63.2)	
**1–2**	46 (50.0)	41 (56,2)	5 (26.3)	
**≥3**	12 (13.0)	10 (13.7)	2 (10.5)	
**Types of comorbidities, n (%)**				
**Obesity**	33 (35.9)	30 (41.1)	3 (15.8)	**0.040**
**Hypertension**	27 (29.3)	24 (32.9)	3 (15.8)	0.145
**Diabetes**	13 (14.1)	11 (15.1)	2 (10.5)	1.000
**Neurological disorders**	8 (8.7)	7 (9.6)	1 (5.3)	0.551
**Asthma**	6 (6.5)	4 (5.5)	2 (10.5)	0.600
**COPD**^**c**^	5 (5.4)	4 (5.5)	1 (5.3)	1.000
**Stage 3 chronic renal failure**	11 (12.0)	8(11.0)	3(15.8)	0.691
**Cardiac failure**	2 (2.2)	1 (1.4)	1 (5.3)	0.372
**Dementia**	1 (1.1)	1 (1.4)	0 (0.0)	1.000
**Cirrhosis**	0 (0.0)	0 (0.0)	0 (0.0)	—
**Symptoms, n (%)**				
**Fever**	81 (88.0)	65 (89.0)	16 (84.2)	0.563
**Cough**	73 (79.3)	57 (78.1)	16 (84.2)	0.557
**Dyspnea**	65 (70.7)	52 (71.2)	13 (68.4)	0.811
**Asthenia**	38 (41.3)	31 (42.5)	7 (36.8)	0.657
**Diarrhea**	31 (33.7)	29 (39.7)	2 (10.5)	**0.027**
**Myalgia**	25 (27.2)	22 (30.1)	3 (15.8)	0.259
**Nausea/vomiting**	23 (25.0)	20 (27.4)	3 (15.8)	0.383
**Headache**	21 (22.8)	16 (21.9)	5 (26.3)	0.684
**Ageusia**	17 (18.5)	11 (15.1)	6 (31.6)	0.099
**Sore throat**	12 (13.0)	9 (12.3)	3 (15.8)	0.707
**Anosmia**	11 (12.0)	8 (11.0)	3 (15.8)	0.691
**CURB 65**^**d**^ **score, n (%)**				0,226
**0–1**	67 (75.3)	50 (71.4)	17 (89.5)	
**2**	16 (18.0)	14 (20.0)	2 (10.5)	
**≥3**	6 (6.7)	6 (8.6)	0 (0.0)	
**Baseline admitting clinical data**				
**Respiratory rate (bpm), mean (SD)**	25.4 (5.5)	26.5 (5.2)	21.6 (4.6)	**0.010**
**Oxygen saturation (%), mean (SD)**	92.1 (2.9)	91.7 (2.8)	93.3 (3.1)	**0.037**
**PaO**_**2**_**/FiO**_**2**_**, median (IQR)**	304 (279–337)	304 (279–337)	337 (304–360)	**0.016**
**Temperature (°C), median (IQR)**	37.4 (36.3–37.9)	37.4 (36.4–37.9)	37.0 (36.0–38.0)	0.313
**SBP**^**e**^ **(mmHg), mean (SD)**	122 (18)	123 (17)	118 (18)	0.232
**DBP**^**f**^ **(mmHg), mean (SD)**	75 (9)	75 (9)	76 (9)	0.850
**Clinical data at the beginning of treatment with remdesivir**				
**Respiratory rate (bpm), mean (SD)**	24.0 (5.3)	24.6 (5.2)	21.1 (4.8)	0.075
**Oxygen saturation (%), mean (SD)**	93.0 (2.5)	92.9 (2.3)	93.2 (3.0)	0.658
**PaO**_**2**_**/FiO**_**2**_**, median (IQR)**	304 (271–340)	291 (268–320)	340 (311–149)	**<0.001**
**Days elapsed between symptom onset and hospital admission, median (IQR)**	6 (4.0–7.0)	6 (4.0–7.5)	4 (2.0–7.0)	0.131
**Days elapsed between PCR/antigen testing and hospital admission, median (IQR)**	3 (1.0–5.3)	4 (1.0–6.0)	3 (0.5–5.0)	0.492
**Days elapsed between symptom onset and beginning of treatment with remdesivir, median (IQR)**	7 (5.0–8.8)	7 (5.0–9.0)	6 (3.0–8.0)	0.187
**Days elapsed between hospital admission and beginning of treatment with remdesivir, median (IQR)**	1 (0.25–1.0)	1 (0–1.0)	1 (0.75–1.21)	0.074

^a^ Patients treated with corticosteroids and/or biologics: remdesivir + corticosteroid bolus + tocilizumab (n = 35, nine of whom received two doses of tocilizumab), remdesivir + corticosteroid bolus (n = 33), remdesivir + corticosteroid bolus + anakinra (n = 3), or remdesivir + tocilizumab (n = 2).

^b^ Patients treated with remdesivir in monotherapy (n = 3) or remdesivir + low-dose corticosteroid therapy (n = 16).

^c^ COPD: chronic obstructive pulmonary disease.

^d^ Data available on 89 patients.

^e^ SBP: systolic blood pressure.

^f^ DBP: diastolic blood pressure.

The most frequent presenting symptoms on admission to the Emergency Department were fever (88%), cough (79.3%), dyspnea (70.7%), and asthenia (41.3%). The patients had a mean RR of 25.4 bpm (SD = 5.5), a mean O_2_Sat of 92.1% (SD = 2.9), and a median PaO_2_/FiO_2_ of 304 (IQR = 279-337).

The median time elapsed between symptom onset and hospital admission was six days (IQR = 4-7), and the median time elapsed between symptom onset and the beginning of the RDV treatment was seven days (IQR = 5-8.8).

Regardingconcomitant treatment, 35 (38%) patients received RDV plus corticosteroid boluses and tocilizumab, three (3%) received RDV associated with corticosteroid boluses and anakinra, 33 (36%) received RDV associated with only corticosteroid boluses, and two (2%) received RDV plus tocilizumab without corticosteroid therapy.

Sixteen (17%) patients were treated with RDV plus low-dose corticosteroid therapy and three (3%) with RDV in monotherapy.

In addition, eight (9%) patients also received plasma from convalescent patients, four cases concomitantly with RDV and bolus corticosteroids ± biological treatment and another four concomitantly with RDV in monotherapy ± low-dose corticosteroid therapy.

Approximately 86% of these patients received the full treatment course (five days of RDV), while 14% (13 patients) discontinued the treatment prematurely due to a worsening of their clinical condition (n = 10, 11%), not being in the viral phase of the disease (n = 1, 1%), a prescription error (n = 1, 1%), or death (n = 1, 1%).

Antibiotic treatment was also administered to 43 (46.7%) patients, 19 (20.6%) of whom received two different antibiotics and five of whom received three antibiotics.

Overall, the mean length of hospital stay was 15 days (SD = 13.4). Nineteen patients (21%) had to be admitted to the ICU for a mean stay of 18 days (SD = 4.2). Regarding the need for ventilatory support, 37% received HFO and 14% NIV, both for a mean of four days. Eleven percent (11%) of cases required IMV for a mean of 22 days (SD = 19.7), and no patients were treated with ECMO.

### Patients’ clinical course

**Fig 1:**
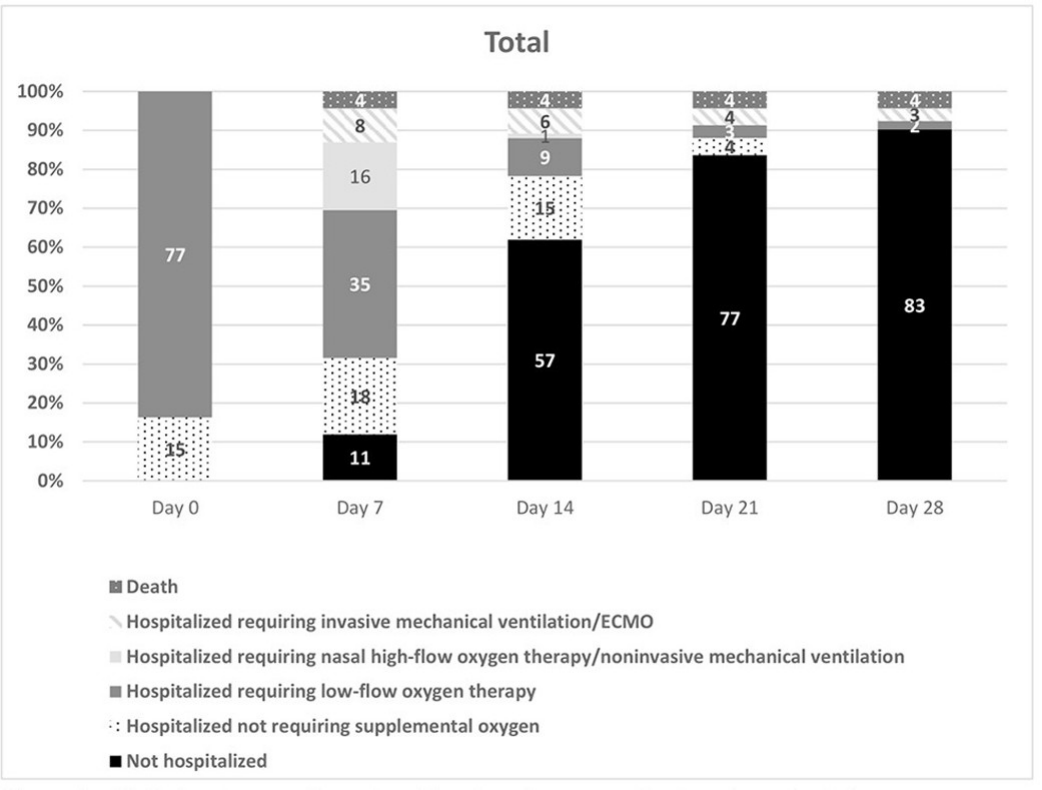
Clinical outcomes based on the six-category ordinal scale endpoints.

At the beginning of the RDV treatment, 77 (84%) patients were receiving LFO and the rest did not require ventilatory support (Fig 1).

On day 7 of the follow-up period, 29 (31%) patients exhibited an improved clinical condition (11 patients had been discharged from the hospital and 18 remained in the hospital without requiring supplemental oxygen therapy). However, 35 (38%) of these patients continued to receive LFO, 16 (17%) received HFO or NIV, and eight (9%) were under IMV. Four deaths were recorded within the first week of starting treatment with RDV. By day 28 of the follow-up period, 83 (90%) patients had been discharged from the hospital, two (2.1%) were receiving LFO, and three (3%) were under IMV. Thus, the number of deaths occurred by day 28 totaled four.

**Fig 2:**
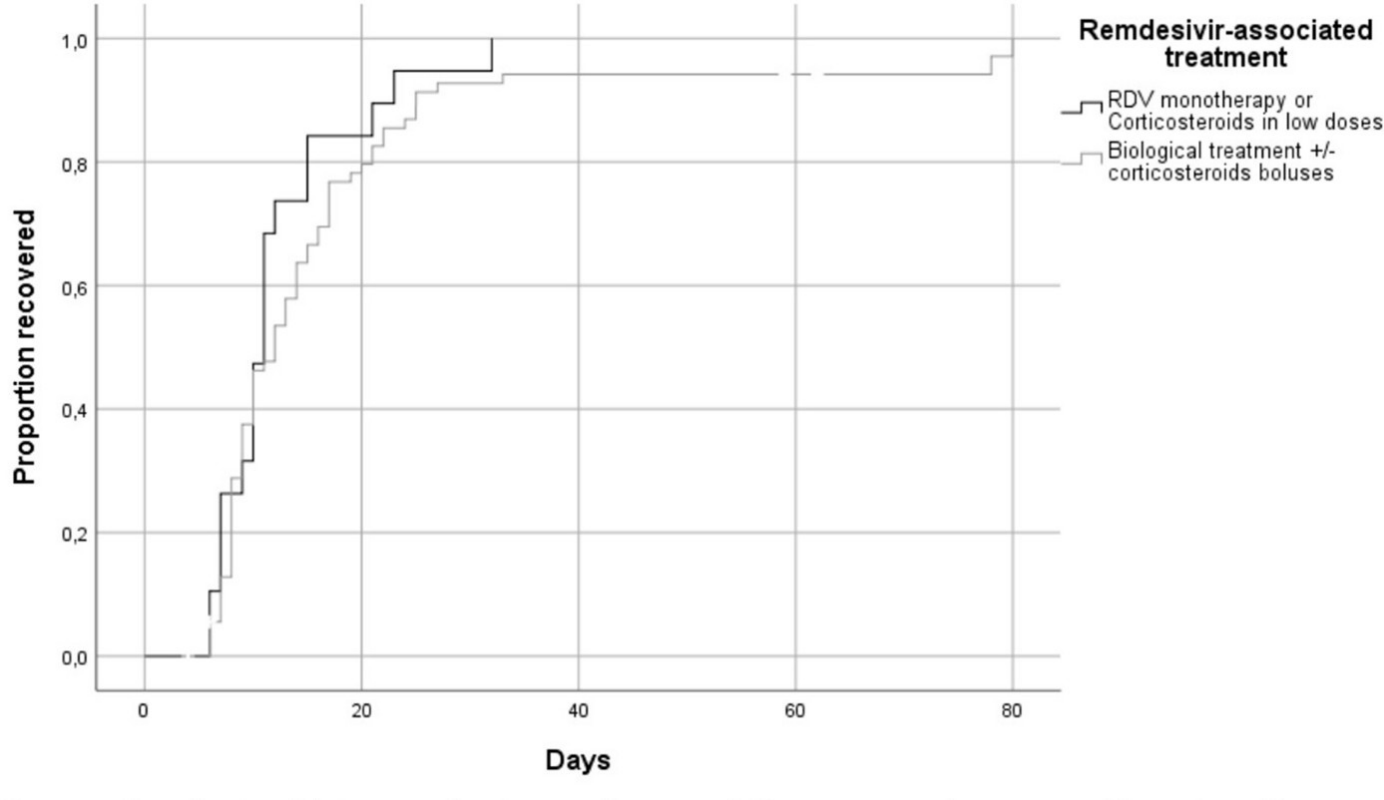
Kaplan-Meier estimates of cumulative recoveries according to the remdesivir-associated treatment.

There were no differences in the recovery time between the patients who received RDV associated with corticosteroid boluses ± biological treatment and those who received RDV in monotherapy or associated with low-dose corticosteroid therapy (median length of hospital stay of 12 vs. 11 days, respectively, *p* = 0.218) (Fig 2).

The Cox regression analysis determined that the likelihood of discharge in patients without hypertension as an associated comorbidity was more than three times higher compared with those with hypertension (hazards ratio [HR] = 3.19, 95% confidence interval [CI] 1.35-7.55; *p* = 0.008). In addition, it also revealed that a higher respiratory rate on admission was correlated with a lower likelihood of hospital discharge (HR = 0.947, 95% CI: 0.89-0.99; *p* = 0.041).

The proportional hazard assumption hold both for hypertension (schoenfeld residuals autocorrelation r = 0.159, p = 0.142) and for respiratory rate (Schoenfeld residuals r = 0.057, p = 0.649).

### Adverse events, complications and mortality

The baseline and follow-up laboratory parameters are outlined in Table 2.

**Table 2:** Laboratory parameters before, during, and after treatment with remdesivir.

	Baseline	Day 3	Day 5	Day 14	Day 28
	n = 92	n = 90	n = 87	n = 30	n = 5
**Hemoglobin**					
No. of patients with available data (%)	92 (100)	90 (100)	87 (100)	28 (93.3)	5 (100)
Mean (SD) (g/dl)	14.0 (1.5)	13.3 (2.1)*	13.7 (1.5)*	12.7 (1.7)*	11.1 (1.9)*
Mean (SD) (mmol/dL)	8.7 (0.9)	8.2 (1.3)*	8.5 (0.9)*	7.9 (1.1)*	6.9 (1.2)*
**Neutrophils**					
No. of patients with available data (%)	92 (100)	90 (100)	87 (100)	28 (93.3)	5 (100)
Mean (SD) (x10^9^/L)	6.1 (5.2)	6.7 (3.8)	6.9 (3.2)	8.0 (4.3)*	10.1 (5.3)
<1 x 10^9^/L, n (%)	1 (1.1)	0 (0)	0 (0)	0 (0)	0 (0)
**Lymphocytes**					
No. of patients with available data (%)	92 (100)	90 (100)	87 (100)	28 (93.3)	5 (100)
Mean (SD) (x10^9^/L)	1.0 (0.5)	1.1 (0.6)*	1.6 (1.0)*	1.8 (1.5)*	2.5 (2.3)
<1 x 10^9^/L, n (%)	49 (53.3)	40 (44.4)	22 (25.3)	7 (25.0)	1 (20.0)
**Platelets**					
No. of patients with available data (%)	92 (100)	90 (100)	87 (100)	28 (93.3)	5 (100)
Mean (SD) (x10^9^/L)	199 (66)	260 (102)*	320 (115)*	235 (93)*	304 (98)
<150 x 10^9^/L, n (%)	22 (23.9)	10 (11.1)	3 (3.4)	5 (17.8)	0 (0)
**D-dimer**					
No. of patients with available data (%)	88 (95.6)	85 (94.4)	83 (95.4)	28 (93.3)	4 (80)
Mean (SD) (μg/L)	770 (710)	746 (678)	1111 (1635)*	1016 (683)	1816 (1214)
>500 μg/L, n (%)	60 (68.2)	47 (55.3)	46 (55.4)	19 (67.8)	4 (100)
**Creatinine**					
No. of patients with available data (%)	92 (100)	90 (100)	87 (100)	30 (100)	5 (100)
Mean (SD) (mg/dl)	0.9 (0.27)	0.8 (0.24)*	0.7 (0.19)*	0.7 (0.28)*	0.7 (0.31)
Mean (SD) (mmol/L)	0.1 (0.02)	0.07 (0.02)*	0.06 (0.02)*	0.06 (0.02)*	0.06 (0.03)
**GFR** ^**a**^					
No. of patients with available data (%)	92 (100)	90 (100)	87 (100)	30 (100)	5 (100)
Mean (SD) (ml/min)	80 (15)	84 (13)*	87 (10)*	83 (15)	80 (21)
≥60 ml/min, n (%)	81 (88.0)	83 (92.2)	83 (95.4)	27 (90.0)	4 (80.0)
59–30 ml/min, n (%)	11 (12.0)	7 (7.8)	4 (4.6)	3 (10.0)	1 (20.0)
<30 ml/min, n (%)	0 (0)	0 (0)	0 (0)	0 (0)	0 (0)
**Bilirubin**					
No. of patients with available data (%)	92 (100)	90 (100)	86 (98.8)	30 (100)	4 (80)
Mean (SD) (mg/dl)	0.6 (0.5)	0.4 (0.2)*	0.4 (0.2)	0.6 (0.2)	0.7 (0.4)
Mean (SD) (μmol/L)	10.3 (8.5)	6.8 (3.4)*	0.4 (3.4)	10.3 (3.4)	12.0 (6.8)
**AST** ^**b**^					
No. of patients with available data (%)	91 (98.9)	90 (100)	87 (100)	29 (96.7)	4 (80)
Mean (SD) (U/L)	54 (65)	42 (29)*	38 (41)*	33 (22)	26 (7)
≥34 U/L, n (%)	54 (59.3)	43 (47.8)	33 (37.9)	10 (34.5)	1 (25.0)
**ALT** ^**c**^					
No. of patients with available data (%)	92 (100)	90 (100)	87 (100)	29 (96.7)	4 (80)
Mean (SD) (U/L)	49 (44)	56 (44)	75 (67)*	75 (60)	66 (36)
≥55 U/L, n (%)	26 (53.3)	37 (41.1)	43 (49.4)	15 (51.7)	2 (50.0)
**LDH** ^**d**^					
No. of patients with available data (%)	88 (95.6)	89 (98.9)	84 (96.5)	29 (96.7)	3 (60)
Mean (SD) (U/L)	352 (117)	330 (116)*	295 (112)*	268 (106)*	227 (84)
>250 U/L, n (%)	79 (89.8)	62 (70.0)	60 (71.4)	13 (44.8)	1 (33.3)
**Albumin**					
No. of patients with available data (%)	89 (96.7)	88 (97.8)	84 (96.5)	29 (96.7)	5 (100)
Mean (SD) (g/L)	39 (4)	36 (4)*	35 (3)*	34 (5)*	32 (4)*
**Troponin**					
No. of patients with available data	90 (97.8)	88 (97.8)	83 (95.4)	29 (96.7)	3 (60)
Mean (SD) (pg/ml)	7.9 (10.7)	9.1 (31.6)	5.7 (12.8)	10.1 (20.9)	18.5 (11.2)
>34.2 pg/ml, n (%)	3 (3.3)	3 (3.4)	2 (2.4)	2 (6.9)	0 (0)
**Ferritin**					
No. of patients with available data	89 (96.7)	90 (100)	86 (98.8)	30 (100)	5 (100)
Mean (SD) (μg/L)	1139 (1782)	997 (1006)	834 (804)	873 (741)	458 (92)
Mean (SD) (nmol/L)	2.6 (4.0)	2.2 (2.3)	1.9 (1.8)	2.0 (1.7)	1.0 (0.2)
>275 μg/L, n (%)	76 (85.4)	76 (84.4)	67 (77.9)	24 (80.0)	5 (100)
**CRP** ^**e**^					
No. of patients with available data	92 (100)	90 (100)	87 (100)	29 (96.7)	5 (100)
Mean (SD) (mg/L)	106.40 (73.33)	66.22 (52.74)*	20.75 (24.89)*	16.45 (38.38)*	32.18 (40.17)
Mean (nmol/L)	1013.3 (698)	630.7 (502.3)*	197.6 (237.0)*	156.7 (365.5)*	306.5 (382.6)
>5 mg/L, n (%)	92 (100)	89 (98.8)	70 (80.4)	10 (34.5)	5 (100)
**PCT** ^**f**^					
No. of patients with available data	90 (97.8)	90 (100)	86 (98.8)	29 (96.7)	4 (80)
Mean (SD) (ng/ml)	0.13 (0.17)	0.12 (0.41)	0.06 (0.19)*	0.07 (0.13)*	0.52 (0.79)
Mean (SD) (					
>0.5 ng/ml, n (%)	3 (3.3)	3 (3.3)	5 (5.8)	1 (3.4)	1 (25.0)

^a^ GFR: glomerular filtration rate.

^b^ AST: aspartate aminotransferase.

^c^ ALT: alanine aminotransferase.

^d^ LDH: lactate dehydrogenase.

^e^ CRP: C-reactive protein.

^f^ PCT: procalcitonin.

At the beginning of treatment with RDV, a considerable proportion of patients presented with elevated serum levels of C-reactive protein (100%), procalcitonin (64.1%), ferritin (88.0%), and lactate dehydrogenase (95.6%). Of these patients, 65.2% had D-dimer levels above the ULN, 12% had a GFR <60 ml/min, 53.3% had ALT levels above the ULN, and 59.3% had AST levels above the ULN before starting treatment with RDV.

Both during and after the administration of RDV, hepatotoxicity was the most frequent adverse event, associated with new-onset hypertransaminasemia in 58 (63%) patients, of whom 10 (10.9%) developed a grade 2-3 event (>3-5 x ULN). This elevation in transaminases was mostly explained by increased ALT levels and exhibited a slow normalization of the mean values throughout the patients’ hospital stay (75 U/L on day 14 and 66 U/L on day 28). Three (3.2%) patients developed new-onset acute renal failure after starting treatment with RDV (grade 2 in one patient). Nausea/vomiting (5.4%) and hypotension (2.2%) were also reported to a lesser extent.

The main complications observed after starting treatment with RDV were nosocomial infections, which were more frequent in the group of patients receiving RDV associated with corticosteroid boluses ± biological treatment (21.9% vs. 5.3%, *p* = 0.181). Of the patients who received RDV + corticosteroid boluses ± biological treatment, 13 (14.1%) presented with ARDS, three (3.3%) with septic shock, and two (2.2%) with thromboembolism.

A total of four patients died during the follow-up period (Table 3). Respiratory failure occurred in all of these cases as a result of the rapid progression of the disease despite the supportive treatment administered in the hospital ward. The age range of these patients was 70-87 years, three of them were women, and all had at least one comorbidity (hypertension in three patients). As for the anti-COVID-19 treatment administered to these patients, all of them had received RDV associated with corticosteroid boluses ± biological treatment.

**Table 3:** Characteristics of the patients who died from COVID-19.

Age	Sex	Comorbidities	Length of stay	Length of ICU stay
87	Female	HT^a^	2	0
84	Male	DM^b^, HT, RF^c^	4	0
74	Female	Obesity, HT	6	0
70	Female	RF	6	0

^a^ HT: hypertension.

^b^ DM: diabetes mellitus.

^c^ RF: grade 3 renal failure (GFR <60 ml/min).

## Discussion

The present study analyzes the clinical course of 92 patients hospitalized for severe COVID-19 pneumonia who had received at least one dose of RDV between 3 July 2020 and 31 October 2020. Eighty-five percent (85%) of the patients completed the full treatment course and 90% of them had been discharged from the hospital by day 28 of the follow-up period after treatment with RDV.

The mean length of hospital stay was 15 days, which is a similar figure to that reported in the pivotal study of RDV [7], and there were no differences in this variable between the severe patients who received RDV associated with corticosteroid boluses ± biological treatment and those severe patients who received RDV in monotherapy or associated with low-dose corticosteroid therapy. This finding suggests that RDV might not be as beneficial in severe patients, in whom a systemic release of inflammatory cytokines has already occurred. However, future studies are necessary to confirm the best COVID-19 patient profile who would benefit from RDV. An evaluation of the clinical course of the study patients based on their score in the modified six-point WHO Ordinal Scale for Clinical Improvement shows that they improved in a similar way to that reported by other authors. A study on the compassionate use of RDV carried out in Italy revealed an improvement 28 days after starting treatment with RDV in 88.2% of patients admitted to the hospital ward versus 38.9% of patients admitted to the ICU [12].

Regarding the need for ventilatory support and admission to the ICU, a Spanish observational study performed in a cohort of patients who were also treated with RDV described a lower percentage of patients requiring IMV (7.3% vs. 11%), but a similar percentage of ICU admissions (19% vs. 21%), compared with our patient population [13]. This difference could be explained by a delayed start of the RDV treatment among the patients included in our study.

The mortality rate in our patient population was 4%, which is a very similar mortality figure to that reported in the cited Spanish observational study [13]. Thus, it can be said that 96% of patients survive a 42-day follow-up period after the first dosing of RDV. García-Vidal et al. observed a higher mortality rate among patients requiring anti-inflammatory treatment, as this is the most severe type of patient population [13]. Because only four patients died during our study’s follow-up period, we were unable to analyze differences in mortality according to the treatment received. All of these patients were part of the subgroup treated with high doses of corticosteroids and biological treatment.

In our patients’ cohort, RDV was practically not used in monotherapy, as corticosteroid and biological therapies were administered concomitantly in most cases due to the severity of the disease.

Therefore, because most patients required concomitant medication, the exclusive effect of this drug on mortality could not be evaluated in our study. Several studies have analyzed the effect of both RDV and corticosteroids on mortality due to COVID-19 [14–16], concluding that RDV does not reduce mortality in patients requiring IMV, while corticosteroids do have a beneficial effect in this type of patient. In fact, the final report of the abovementioned pivotal clinical trial highlights the benefits associated with the use of RDV in patients requiring oxygen therapy but not IMV [7].

The patients of our study cohort evidenced clinical manifestations of COVID-19 (i.e., fever, cough, dyspnea, and asthenia) [1]. As observed in other studies, 65% of them had at least one comorbidity, the most prevalent being obesity, hypertension, and diabetes mellitus [17, 18]. Obesity had already been linked to both an increased risk of hospitalization and an unfavorable outcome in previous studies, and hypertension had also been identified as a predictor of increased mortality.

In fact, our analysis identified both high respiratory rate and hypertension as factors with a statistically significant correlation with a lower likelihood of hospital discharge. This finding is relevant, as contributes to defining a risk COVID-19 patient profile. In fact, given the long course of this pandemic and the high pressure that is putting on health systems, an efficient stratification of patients and use of resources is necessary.

Short-time variations in neutrophil-to-lymphocyte ratio and urea-to-creatinine ratio have been proposed as early predictors of clinical deterioration [19]. Our study complements this finding, by showing that hypertension and a high RR could also help to identify cases of worsen disease. Therefore, if confirmed prospectively, the evaluation of all this markers before the onset of severe manifestations should be recommended, helping physicians to stratify patients and to initiate the most appropriate management.

Moreover, the identification of a risk COVID-19 profile is interesting to focus future research in these patients, as key genes and pathways of COVID-19 pathogenesis could be revealed, contributing to the development of new potential drugs. Having assessed the safety of RDV, the most frequent adverse event observed in our study was an increase in the levels of hepatic enzymes, which normalized subsequently. Similar findings were reported in the compassionate use study [12]. Given that other adverse events reported in several studies [6–9, 20], such as nausea/vomiting or hypotension associated with RDV, were recorded to a lesser extent in the CMRs of our patient population, they could not be compared with those reported in other works.

There was a greater incidence of nosocomial infections among patients who received RDV associated with corticosteroid boluses ± biological treatment compared with those who received RDV in monotherapy or associated with low-dose corticosteroid therapy, although the difference was not statistically significant. This could be explained by the fact that patients treated with tocilizumab and high doses of corticosteroids may be more immunosuppressed, as demonstrated in other studies [13, 20, 21]. Forty percent (40%) of the patients received antibiotic treatment despite only 18% of them having a positive culture, which suggests that antimicrobials may have been overused during this pandemic in patients in whom bacterial coinfection is not as prevalent. A call for caution in the prophylactic use of antibiotics is recommended to prevent the emergence of bacterial resistance.

The emerging SARS-CoV-2 pandemic has led to an increase in hospital pressure, with effective treatment being required to mitigate disease progression to severe COVID-19 pneumonia.

There is still uncertainty concerning the best treatment for COVID-19 pneumonia. To date, the only treatment that has proven to be effective is dexamethasone at a dosage of 6 mg/day [15]. As for RDV, there is currently controversy in relation to the results obtained in the different studies carried out with this drug.

The first clinical trial with RDV was conducted in ten hospitals in Wuhan, China, where a ten-day course of this drug was administered to patients presenting with severe COVID-19 pneumonia, observing no significant decrease in the risk of death at 28 days of follow-up [8].

The final report of the Adaptive COVID-19 Treatment Trial 1 (ACTT-1) study described shorter recovery times in patients hospitalized for COVID-19 pneumonia who were treated with RDV compared with those who received a placebo [7]. However, the WHO-promoted SOLIDARITY trial reported no significant differences in terms of mortality, the need for mechanical ventilation, or the length of hospital stay between patients who received RDV and those who received a placebo [15].

Hence, further studies should be carried out to determine the effect of RDV in real-life conditions, as this drug continues to be in the current COVID-19 treatment protocols [22].Vaccination has been one intervention strategy to the control of transmission that the majority of countries have activated. Nevertheless, spread of COVID-19 continues, since there is a population that has not been vaccinated or has received the vaccine in an incomplete pattern. Negative serology can also occur in immunocompromised persons, who may have no response to the vaccine. As a treatment option to these patients, anti-SARS-CoV-2 neutralizing antibodies have recently been approved in Europe [23, 24]. However, high viral replication in immunodeficiency diseases may persist long-time [25], and RDV could be a beneficial antiviral in this context. Therefore, future comparative studies of RDV vs monoclonal antibodies or regarding the combination of both therapeutic strategies would be interesting.

One of the limitations of this study was its observational design without a control group, owing to which we were unable to estimate the effectiveness of RDV in the treated patients. However, the main objective of this study was not to analyze the drug’s effectiveness, but rather the clinical course of the patients treated with RDV in a reference hospital. All patients were treated according to clinical practice, following the protocols available for the treatment of COVID-19, and no exclusion criteria was applied by the investigators. Therefore, the results of this study can be generalized to severe COVID-19 patients attended in actual practice. Exception are pregnant women and children under 12 years-old, as there were no cases treated with RDV during the study period. Another limitation was its retrospective nature, as adverse events may be underreported in this type of study. However, the laboratory parameters collected from the patients’ CMRs were monitored to minimize this issue. In addition, a larger number of patients in the study cohort would have favored a greater statistical power in the subgroup and multivariate analyses. Despite the foregoing, our study allowed us to identify one of the most prevalent comorbidities (i.e., hypertension) as a prognostic factor for a prolonged hospital stay.

Thus, this study provides real-life information on the clinical course of a cohort of severe COVID-19 patients treated with RDV during the first months of this drug availability at a worldwide level. After multiple outbreaks of COVID-19 there is not still an effective treatment, while this disease continues to threaten the overall health. Many different drugs were used during the first wave of the pandemic [26], but RDV is the only one that currently remains in the therapeutic arsenal of the disease. Therefore, the high survival rate and the drug safety observed in this study provide reassurance for the future use of RDV throughout the pandemic. This study may contribute to evaluate the current therapeutic role of RDV in clinical practice, which it is essential for making policy decisions in an emerging severe disease.

## Conclusion

Patients with severe COVID-19 pneumonia who have received treatment with RDV require relatively prolonged hospital stays, many with a need for ventilatory support and, in a considerable proportion of cases, admission to the ICU. However, the observed survival rate is high and approximately 90% of patients are discharged from the hospital. RDV can be considered a well-tolerated drug with no associated adverse events that could have an impact on treatment continuity. Hypertension and high RR have been identified as variables significantly correlated with a need for prolonged hospital stays. This finding may contribute to identify patients in risk of unfavourable evolution, who may beneficiate for a close monitoring and early care support.

## Supporting information

S1 File (XLSX)
